# Patient expectations of fair complaint handling in hospitals: empirical data

**DOI:** 10.1186/1472-6963-6-106

**Published:** 2006-08-18

**Authors:** Roland D Friele, Emmy M Sluijs

**Affiliations:** 1 NIVEL, Netherlands institute for Health Services Research, PO Box 1568, 3500 BN Utrecht, The Netherlands

## Abstract

**Background:**

A common finding in several studies is patients' dissatisfaction with complaint handling in health care. The reasons why are for the greater part unknown. The key to an answer may be found in a better understanding of patients' expectations. We investigated patients' expectations of complaint handling in hospitals.

**Methods:**

Subjects were patients who had lodged a complaint at the complaint committees of 74 hospitals in the Netherlands. A total of 424 patients (response 75%) completed a written questionnaire at the start of the complaint procedures. Derived from justice theory, we asked what they expected from fair procedures, fair communication and fair outcome of complaint handling.

**Results:**

The predominant reason for complainants to lodge a complaint was to prevent the incident from happening again. Complainants expected fair procedures from the complaint committee, in particular an impartial position. This was most important to 87% of the complainants. They also expected to be treated respectfully. Furthermore, they expected the hospital and the professional involved to respond to their complaint. A change in hospital performances was the most wanted outcome of complaint handling, according to 79% of the complainants. They also expected disclosure from the professionals. Professionals should admit a mistake when it had occurred. More complainants (65%) considered it most important to get an explanation than an apology (41%). Only 32% of complainants expected the professional to make an effort to restore the doctor-patient relationship. A minority of complainants (7%) wanted financial compensation.

**Conclusion:**

Nearly all complainants want to prevent the incident from happening again, not out of pure altruism, but in order to restore their sense of justice. We conclude that complaint handling that does not allow for change is unlikely to meet patients' expectations. Secondly, complaint handling should not be left exclusively to complaint committees, the responses of hospital and professionals are indispensable.

## Background

### Introduction

Many patients appear to be dissatisfied with legal litigation procedures [1] and also with non-legal complaints handling in hospitals [2-4]. This phenomenon is not well understood.

Little is known on patients' motives to lodge complaints and about patients' needs and expectations regarding the complaint handling procedures in hospitals [3,5]. This article tries to fill the gap. In this study, we gathered empirical data from over 400 patients when they initiated a complaint against the hospital. The aim was to find out what they expected from the complaints handling process and which aims they wanted to achieve with the complaint.

### Complainants' dissatisfaction as motive for the study

Complainants dissatisfaction is a common finding in many studies [2-4]. Daniel evaluated the experiences of 290 patients whose complaints were finalised by the Health Care Complaint Commission of New South Wales (HCCC). Nearly two thirds of the patients (61%) were dissatisfied with the complaint handling by the time the complaint file was closed [2]. Many respondents remained angry and most wanted stern measures to be taken. Indeed, if strong action had been taken against the doctor, satisfaction was significantly more likely. Similar results of dissatisfied complainants were found in our former study on Complaints Commissions in hospitals[4]. Of the professionals who gave cause to the complaint, two third were satisfied with the complaint handling. In contrast, only one third of the complainants were satisfied with how their complaint was handled. Particularly puzzling was the finding that one third of the complainants remained dissatisfied despite the fact that their complaint was judged as founded. These complainants were formally put in the right, suggesting that their aims had been achieved. This disappointing result was the immediate cause of a sequel study into patients' expectations reported in this article [6].

The dissatisfaction was contrary to all expectations because – just as in other countries [2,7-9] – substantial improvements had been made in the complaint handling system in the mid-nineties [10]. The 'Clients' Right of Complaint Act' was enacted in 1995 in the Netherlands. The act legally obliged all health care institutions and professionals to install easily accessible independent complaint committees. These commissions were supposed to fill the gap between informal complaint handling (aimed at support and mediation by patient service offices or complaint officers) and formal complaint bodies as the Disciplinary Boards and the Civil or Criminal Court [10]. At the evaluation of the Act in 1999 most hospitals appeared to comply with the legal obligations [4]. It turned out that the first aim of the Act – to warrant easily accessible non-legal complaint facilities for patients – had been achieved. The second aim however – to restore patients' satisfaction with and trust and confidence in health care – was not attained. The Minister of Health suggested that an explanation might perhaps be found in the discrepancy between complainants' expectations and experiences [5].

### Patients' motives and expectations

Some research has been done on patients' motives to complain [11,12]. Bark et al. analysed 491 questionnaires of complainants whose complaint files were in progress or already closed. All complainants reported a combination of reasons for their complaint. The majority of the patients (90%) wanted to prevent a similar incident to avoid others having to go through a similar experience. The patients (80%) also wanted staff to be aware of what had happened and the effect it had had on the patient. Many patients reported emotional pain and suffering as a result of the incident and strong feelings of anger, distress, worry and depression.

One in four patients wanted an explanation and half of the patients wanted an apology. A minority of complainants (9%) wanted compensation [11,12].

Vincent surveyed 227 patients who had called in medical negligence solicitors: two thirds of the patients wanted financial compensation[12]. Besides financial compensation, the patients reported a number of other reasons for litigation. About 90% of the patients had marked the following (closed) items as reason for litigation: 'that it would not happen to anyone else' and 'I wanted an explanation' and, 'I wanted doctors to realise what they had done'. Thus, financial compensation may be a reason to lodge a complaint against a doctor, it will not be the only reason [12,13]. Many patients say they want to prevent recurrence of the incident by suing doctors or lodging a complaint. Triemstra et.al. suggest that altruism might be a main motive to complain [14]. This, however, does not explain why patients may be dissatisfied or even angry about complaint handling. Are they dissatisfied with the procedures or with the outcome (for example apology, punishment, or compensation)? According to Daniel, understanding what patients expect may obviate some of the difficulties and disappointments revealed by complaint surveys [2].

It should be noted that particularly little is known about patients' needs and expectations at the *start *of the complaint procedures. The figures so far reflect patients' feelings afterwards, thus after the complaints are closed. Looking back, patients could have changed their expectations. That is why this study inquires into patients needs and expectations at the *start *of the complaint handling procedures. Our research questions are as follows.

### Research questions

1. Patients' expectations: what do patients expect from the complaint handling in hospitals?

2 Patients' motives: which aims are they trying to achieve in lodging a complaint?

3 Are patients' expectations related to patients' characteristics or the nature of the complaint?

Patients' expectations are their beliefs about how the parties involved should or will perform [5]. There is a conceptual distinction between *will- *and *should *expectations. *Will*-expectations correspond with what patients believe will happen. Meeting will-expectations does not automatically yield satisfaction. If will-expectations are low – for example 'they will not respond' – satisfaction is most unlikely. *Should*-expectations represent 'what ought to happen', it is a normative standard [5]. In our study we focus on what according to the patients should happen and which aims they wanted to achieve.

We investigated what patients expected from the complaint committees and which response they expected from the other two parties involved, the (accused) professional and the hospital(management). Basic to our study is the question: what would be fair solutions in the complainants' eyes?

Further specification of this question was based on 'justice' theory [15]. According to justice theory, people expect a fair handling of the whole complaint handling process, which means a) fair procedures, b) fair interpersonal communication and, c) a fair outcome. These three dimensions correspond with the main concepts in justice theory: procedural justice (dealing with decision making procedures), interactional justice (dealing with interpersonal behaviour) and distributive justice (dealing with decision outcomes) [15]. These dimensions were used to investigate the complainants' expectations about the fairness of the complaint handling process.

## Methods

### Setting

All 97 academic and general hospitals in the Netherlands and their complaint committees were invited to participate in the study.

### Subjects

All patients who had lodged a complaint at the complaint committee of the hospital during the first six months of 2003.

### Procedures

- The Complaint Committees of each hospital received our invitational letter and the privacy protocol. They were asked to address our package (with questionnaire, letter and return envelop) to all the complainants without selection.

- Complainants received the questionnaire immediately after their complaint had been received.

- The completed questionnaires were sent back directly and anonymously to our research institute NIVEL.

### Informed consent procedure

An explicit informed consent procedure was followed: explaining about the aim of the study and making clear that participation or non-participation in the study would not have any impact on the treatment of the complaint. This was achieved through the following procedures:

- The complaint committee and the hospital employees did not see the complainant's questionnaire. They did not know which complainants had completed the questionnaire,

- It was explained to the complainants (in a letter) that they were entirely free in their decision whether or not to complete the questionnaire. No reminder would follow and their response would be treated confidentially.

- The letter also explained that patients' responses to the questionnaire would and could have no bearing on the conduct or outcome of the complaint procedures.

- A written privacy protocol was used to process the data. This protocol had been sent to all the Complaint Committees. The professionals involved and the hospital management were also given this protocol, when they asked for it.

No formal approval from an ethics committee was sought, since this study is not an experiment, the task that is required from respondents is not invasive (people are asked to fill in a questionnaire, not more) and, finally, the issue of the study is not 'medical care', but the way hospitals react on complaints. It was therefore concluded that no formal ethical approval was required. This was communicated to the participating hospitals before the start of the study.

### Development of the questionnaire

The questionnaire was based on open interviews with a total of 15 complainants whose complaints were finalised (11 interviews with individual complainants and one focus group meeting with four other complainants).

We listened carefully to patients' stories and tried to understand why patients were dissatisfied with the complaint committee, despite the fact that their complaint was judged as founded.

The interviewees mentioned a number of important aspects of complaint handling [6]. The way the hospital board and the professionals react to their complaint is important to them, as important as the reactions from the complaint committees. The complainants considered the hospital's and the doctor's responses to be the ultimate outcome of their complaint handling. That is why the questionnaire not only focuses on the complaint committee, but also on the reactions of the hospital board and the professionals. In the questionnaire, only those topics were included that were most important according to the interviewees. These are reported in the tables 1 to 4.

### Content of the questionnaire

The questionnaire has been structured around the three dimensions of the fairness theory: procedures, communication and outcome. Besides demographic items, the main issues in the questionnaire are:

- The nature of the complaint (4 items)

- What do you expect from the complaint committee with respect to the *interpersonal communication *(5 items) and the *procedures and outcome *(7 items)

- What do you expect from the hospital board with respect to *interpersonal communication *and the *outcome*? (4 items).

- What do you expect from the professional(s) who gave cause to the complaint with respect to *interpersonal communication *and the *outcome *(5 items)?

- What were your main motives to lodge a complaint? (6 items)

Nearly all items are closed questions with four response categories, namely: this issue is for me: 1) not important, 2) important, 3) very important 4) extremely or most important. Much room was left in the questionnaire for the patients to give explanations in their own words.

### Analysis

The focus of the first two research questions is descriptive. Hence, results will be presented using descriptive statistics. To answer the third question, complainants' characteristics (age, gender, education) and information on the nature of the complaint will be related to expectations. Four expectation-scales were constructed and their reliability was tested: Expectations regarding the committee's interpersonal communication (5 items, α = 0.74), Expectations regarding the committee's procedures and outcome (7 items, α = 0.74), Expectations regarding the interpersonal communication and the outcome of the hospital board (4 items, α = 0,62) and Expectations regarding the professional's interpersonal communication and outcome (5 items, α = 0,69). Reliability was considered at least acceptable to allow for further analysis.

### Respondents

Of the 97 Complaint Committees, 76 participated in the study (response 76/97 = 78%). Reasons for not participating differed, for example committees 'would protect the complainants', 'would not bother the complainants', 'received too few complaints per year', 'temporal vacancy of the committee's secretary' or 'there were objections of the hospital board'.

A questionnaire was sent by the Complaint Committees to 563 complainants. Of them, 424 complainants returned the questionnaire to our institute (response 563/424 = 75%). Non-response analyses were not possible, because those data were not at our disposal.

## Results

### Participants

More women than men completed the questionnaire: 67% of the 424 respondents is female and 33% a male. The respondents represent a relatively high educational level: 40% has a higher or academic education.

The event which gave cause to the complaint usually concerned several aspects. According to the respondents, over two thirds of the complaints (68%) concern clinical conduct of medical specialists, frequently in combination with shortcomings in relational conduct or shortcomings in the information provided by the professional. Less frequently are nursing care (23%) and/or organisational incidents (37%) the cause of the complaint. A minority of complaints (9%) exclusively concerns the doctor-patient communication.

The majority of patients considered the incident as very serious and many reported detrimental consequences due to the incident (suffering, pain or health damage and feelings of anger, distress, anxiety or depression). A minority of the complainants (7%) had filed a claim for financial compensation. Table 1 shows patients' expectations of the *procedural *conduct and *outcome *of the complaint committee.

Because nearly all aspects of the procedures appear to be important to the complainants, we focus on the most important ones. The complaint committee should recommend the hospital to change things. This is considered to be of the utmost importance by the great majority of complainants (89%). They also expect the committee to make an inquiry into the incident and to deliver a grounded judgement about the validity of the complaint. To give such a judgement is a legal task of complaint committees. Many, but not all complainants want to meet the committee's members personally, to tell them their own story about what exactly has happened in the hospital. A minority (18%) does not prefer a face to face meeting with the committee. They seem to prefer a settlement in writing. The complainants expect to receive clear information about the committee' s procedures and a swift response. Although important, these latter two procedural affairs seem to be less important to the complainants than the interpersonal conduct of the complaint committee, as Table 2 shows.

Regarding the interpersonal conduct of the complaint committee the majority of the complainants (87%) consider the impartiality or independent position of the utmost importance. Obviously, people expect to be treated respectfully by the members of the committee. They don't want to be treated as 'trouble makers'. It appears that more complainants want the committee to show understanding or concern, but relatively less complainants expect expressions of pity or compassion; it seems that they don't want to be treated pathetically. Table 3 shows patients' expectation of the hospital board.

The hospital board should discuss the incident with the employees and/or with the department which caused the incident or who were involved in what happened. They should be confronted with the complaint. However, punishment is not important to 34% of the complainants; they are not aiming at penalisation of the professionals involved. The majority of the complainants want correcting measures to be taken by the hospital board. The complainants expect to be informed about the fact *that *correcting measures have been taken and preferably also *which *measures were taken in response to the complaint. Complainants appear to strive for alterations in the hospital. Patients' expectations of the professionals involved are shown in table 4.

The professionals should admit a mistake when it has occurred. Such an admission is most important to many complainants (84%). Fewer complainants consider an apology offered to them as most important (41%). An apology is even considered to be unimportant to about one fourth of the complainants. They want an explanation about how the incident could have happened. They want to 'know'. Most complainants do not expect expressions of sympathy from the professionals and a noticeable number of complainants (53%) are not striving for regaining a (good) relationship with the professionals. Although the recovery of the doctor-patient relationship was one of the aims of the new complaint act.

Subsequently, we studied the relationship between complainants' characteristics (age, gender, education) and the nature of the complaint on the one hand and the scores on the four expectation-scales on the other hand. Women express a higher demand for the committee's interpersonal communication: results show gender to be related to one scale: the committee's interpersonal communication (p = 0.001; F-test). Likewise lower educated complainants express higher demands for the committee and the professionals, as is shown by the correlation of educational level with the scores on three scales: the committee's interpersonal communication (p = 0.001;correlation = -.16), the committee's procedures and outcome (p = 0.006; correlation = -.14) and the professional's communication and outcome (p = 0.019; correlation = -.13). Younger people express higher demand on the hospital board, as age was correlated to one scale: the hospital board's communication and outcome (p = 0.001;correlation = -17). No difference in expressed demands was found between complaints with a complaint that concerned themselves, or a complaint that concerned another person (e.g. child or partner). When complainants experienced serious harm, they express strong expectations on all issues: a significant relationship (correlations varying from .17 to .22) was found between all four scales and the degree to which complainants had experienced serious harm.

For nearly all patients, the main reason to lodge a complaint was to prevent the incident from happening again to other patients. Many complainants were also led by a sense of duty or justice: 'this should never happen again'. According to more than two thirds of the complainants, what had happened went against their sense of justice. Most people also felt it their duty to lodge a complaint. Many complainants were severely affected and some of them wrote that they 'owed it to the person who died'.

Our results correspond to former studies, in so far that preventing the incident from happening again appears to be a main reason to lodge (formal) complaints. However, complainants' motives are not mere or pure altruism, as the interviews with complainants showed. Illustrations of patients' motives are given below in the complainants' own words (Table 6).

These motives of the complainants illustrate that many patients seem to be driven by strong feelings of justice and duty. Their motives also reflect concern for other patients (altruism), but the complainants' motives seem to express a more general feeling that "something must change".

## Discussion

### Aim and relevance of this study

This study investigates patients' expectations about complaint handling in hospitals. Such knowledge was deemed to be necessary because a number of surveys had indicated prevailing dissatisfaction among complainants about the way in which their complaints had been handled. These findings were surprising and disappointing, because substantial improvements in the complaint handling systems had been made in many countries in the nineties. These improvements were in concordance with a more general movement of the last decades towards strengthening the position of patients and reflected the increased concern for patients' rights in health care.

The rationale of our study is the central idea that complaint handling should meet patients' expectations to be effective and satisfying to complainants. That is why we investigated what patients expected when they lodged a complaint at the hospital's complaint committee. A total of 424 patients responded (75%); relatively many of them (40%) were highly educated. This study is unique in that patients' expectations were explored at the very start of the complaint handling process.

### Complainants' expectations

The complainants appeared to be rather unanimous in their opinions about a fair complaint handling process. According to the vast majority of the complainants, the complaint committee should investigate the incident, give a motivated adjudication about the validity of the complaint and recommend the hospital to change things. The complainants expect to be treated respectfully by the members of the complaint committee and an impartial attitude of the committee is very important to them. An independent position of the complaint committee is supposed to contribute to the complainants' confidence in a fair complaint process.

As important as the complaint committees' conduct are the reactions of the hospital board and the professionals to the complaint. The hospital board should discuss the complaint with the staff and should inform the complainant that corrective measures have been taken as a result of the complaint. Complainants feel that the professionals should explain how the incident could have happened, but above all, they should admit a mistake when it has occurred.

We must conclude that the reactions of all the three parties involved in the complaint handling are of equal importance to the complainants. Although the most severely affected complainants have the highest expectations of complaint handling, these expectations concern all the three parties involved, the committee, the hospital and the doctor. These patients don't want different things, they want the same, but more intensely.

### Fairness of the process, the communication and the outcome

The complainants' expectations can be related to the fairness theory. This theory predicts that complaints handling will be satisfactorily to patients if the three dimensions of the process are evaluated as being fair: fair procedures, fair communication and fair outcome. Relating these three 'fairnesses' to the three parties involved, some emphasis can be seen. Procedural fairness is expected in particular from the complaint committee. Fairness regarding outcome appears in particular to be expected from the hospital board (a change in hospital's performances). The communicational or interpersonal fairness seems largely to be expected from the complaint committee (respectful conduct) as well as from the professionals involved (explanations and acknowledgement). We must conclude that a complaint system that only focuses on complaint committees and complaint procedures is incomplete in the complainants' views. This is an important lesson to be learned from this study. It may be assumed that complainants will be more satisfied with the complaint handling process when all three dimensions are fair in the complainants views: procedures, communication as well as outcome. This hypothesis needs to be tested in future research. Meanwhile, hospitals could evaluate their complaints processes thoroughly in the light of the findings of this study.

### Meeting patients' expectations

Meeting patients' expectations may be more within reach of the hospital and the complaint committee than of the professionals involved. Professionals will face barriers which prevent them from meeting patients' strong wishes for disclosure of medical errors. According to Gallagher et al., many current institutional policies about disclosing medical errors instruct physicians not to discuss why an error has happened in a way that could imply fault[16]. Although patients and physicians agree that such disclosure is ethically imperative, many physicians said that fear for litigation limited them in what they tell patients about errors. Furthermore, physicians themselves experienced powerful emotions following medical errors and complaints, resulting in diminished self-confidence or anxiety regarding their reputation. Gallagher concludes that both patients and physicians have unmet needs following errors, and better institutional support for caregivers involved in errors would help them focus their attention on the affected patient[16].

### Limitations of the study: cultural differences

The main limitation of our study concerns the generalisation of the results. Cultural differences between countries – but perhaps more between continents – may play a role in the complainants' expectations and their preferred way of complaint handling. In most European countries, the emphasis is on mediation and finding non-legal solutions for complaints about health care[17]. Traditionally, in these regions, a 'claim'-culture does not exit. In contrast, in the United States and United Kingdom, there appears to be a strong and increasing tendency to claim for financial compensation and to call in medical negligence solicitors to sue doctors[18]. In our study, only a minority of the complainants (7%) had made a claim for financial compensation. Therefore, our results may not be valid worldwide. However, Vincent [12] found similar motives to sue a doctor as we found motives to complain. They also found as the main motive to prevent the incident from happening again. Maybe the cultural differences are not so dominant after all when it comes to health care complaints.

### Complainants' motives to complain

In concordance with other studies, we found as a main motive for complainants to initiate a complaint that they wanted 'to prevent the incident happening to others'. It is tempting to take this statement literally and to assume that complainants' main motive is to protect other patients against such an incident. However, some nuances are to be made regarding this supposed 'altruism' of complainants. A better understanding of their motives was derived from patients' explanations during the face to face interviews in preparation of this study. Complainants motives reflected general feelings of injustice and wrongness. The majority of the complainants felt it as their duty to complain because what had happened went against their sense of justice. The incident had caused severe embarrassment and they reacted out of a fundamental feeling that something had gone wrong in the order of things which had to be put into the right. In our view, patients' main motives to initiate a complaint seems to be a general feeling that 'this should never happen again' to restore their sense of justice.

## Conclusion

The results of our studies indicate that nearly all complainants strive to realise a change in performance as response to their complaint. Perhaps, such a change may be felt as the ultimate acknowledgement and may help to restore the complainant's sense of justice. Our final conclusions are twofold. Firstly, complaint handling should not be left exclusively to complaint committees, the complaint process should encompass all parties involved. Secondly, complaint handling that does not allow for change is unlikely to meet patients' expectations.

## Competing interests

The author(s) declare that they have no competing interests.

## Authors' contributions

RF conceived the study, participated in its design and realization and helped to draft the manuscript. ES carried out the study and drafted the manuscript. All authors read and approved the final manuscript.

## Pre-publication history

The pre-publication history for this paper can be accessed here:



## Figures and Tables

**Table 1:** Patient expectations of *procedures and outcome *of the complaint committee, expressed in percentage of patients (N = 424).

Importance	not important	important	very- and most important
***Procedures: ***	%	%	%
*The complaint committee should*:			
- recommend the hospital to change things	1	6	89
- give an adjudication about the complaint's validity	2	13	82
- give an explaining rationale of the adjudication	3	14	79
- investigate the incident	3	15	78
- give clear information about the complaint procedures	2	33	59
- give me the opportunity to tell what happened personally	18	21	53
- respond swiftly	7	44	44

*) missing values vary from 3% to 9% per item

**Table 2:** Patient expectations of *interpersonal *conduct of the complaint committee, expressed in percentage of patients (N = 424).

Importance	not important	important	very- and most important
*The complaint committee should*:	%	%	%
- take an impartial attitude and position	1	8	87
- treat me respectfully	2	12	82
- show understanding for my experiences	6	18	73
- listen to my own story of what happened	6	15	71
- show sympathy for what I went through	23	25	47

*) missing values vary from 3% to 7% per item

**Table 3:** Patient expectations of the *conduct *of the hospital board and the *outcome *in response to the complaint, expressed in percentage of patients (N = 424).

Importance	not important	important	very and most important
*The hospital board should:*	%	%	%
- make the complaint to be discussed with the employees or department involved	2	12	80
- let me know **that **corrective measures have been taken	5	11	79
- let me know **which **corrective measures were taken	12	14	68
- take punitive measures when mistakes were made	34	17	39

*) missing values vary from 5% to 10% per item

**Table 4:** Patient expectations of the *conduct *of the medical professional subject to the complaint and of the *outcome*, expressed in percentage of patients (N = 424).

Importance	not important	important	very and most important
*The professional subject to the complaint should:*	%	%	%
- admit a mistake when it has occurred	2	7	84
- explain how the incident could have happened	9	14	65
- offer an apology	24	22	41
- show sympathy for what I went through	29	21	38
- make an effort to recover our relationship	53	17	15

*) missing values vary from 8% to 15% per item

**Table 5:** Reasons for patients to lodge a complaint expressed in percentage of complainants (N = 424).

Importance	not important	important	very important + most important
*Reasons to complain*	%	%	%
- I want to prevent the incident happening to others	1	4	94
- I want the complaint to be known at a higher level	7	14	75
- What has happened goes against my sense of justice	11	13	70
- I feel it as my duty to lodge a complaint	12	15	68
- I want to prevent the incident happening to me again	14	13	67
- I want to prevent the incident being kept private	20	22	52

*) missing values vary from 2% tot 6% per item

**Table 6:** Complainants motives to lodge a complaint.

- My first motive is to attack injustice.
- 'It is your duty. This should not happen to anyone else. Something has to change'.
- 'My aim is that something will change. I thought that the committee would be able to achieve more things than an individual patient'.
- 'I want justice to be done. I owe it to my deceased husband. I must avail of all manners to raise the issue. Otherwise I would regret it my whole life'.
- 'I have lodged a complaint despite the apology of the doctor. That is not enough. I don't let it down. I have suffered a lot last weeks'.
- 'My motive is to prevent it happen in the future. People should learn from it. Something must change'.
- My motive was to be taken seriously. I had suffered much pain and anxiety and the doctor had not taken it seriously.
- You should make known that things go wrong. That is your duty.
